# Age-period-cohort analysis of lung cancer mortality in China and Australia from 1990 to 2019

**DOI:** 10.1038/s41598-022-12483-z

**Published:** 2022-05-19

**Authors:** Ning Wang, Zhiwei Xu, Chi-Wai Lui, Baohua Wang, Wenbiao Hu, Jing Wu

**Affiliations:** 1 National Center for Chronic and Noncommunicable Disease Control and Prevention, Chinese Center for Disease Control and Prevention, Beijing, 100050 China; 2 School of Public Health and Social Work, Queensland University of Technology, Brisbane, QLD 4059 Australia; 3 School of Public Health, Faculty of Medicine, University of Queensland, Brisbane, QLD Australia

**Keywords:** Cancer epidemiology, Lung cancer, Cancer

## Abstract

Lung cancer (LC) is the leading cause of cancer death in China and Australia, the countries with different socioenvironmental contexts in the Western Pacific Region. Comparing the age-period-cohort effect on LC mortality (LCM) between the two countries can help plan interventions and draw lessons for countries in the region. We collected LCM estimates between 1990 and 2019 from the GBD 2019. Age-period-cohort modelling was applied to compute the net drift, local drift, cross-sectional age curve, longitudinal age curve, and the rate ratios (RRs) of period and cohort. China had a higher LC age-standardized mortality rate than Australia in 2019 (men: 58.10 [95% uncertainty interval (UI): 46.53, 70.89] *vs.* 30.13 [95% UI: 27.88, 32.31]/100,000 population; women: 22.86 [95% UI: 18.52, 27.52] *vs.* 17.80 [95% UI: 15.93, 19.34]/100,000 population). Period and cohort effects on LCM improved more markedly among Australian men (RR for period effect, from 1.47 [95% confidence interval (CI) 1.41, 1.53] to 0.79 [95% CI 0.75, 0.84]; RR for cohort effect, from 2.56 [95% CI 2.44, 2.68] to 0.36 [95% CI 0.11, 1.18]) and Chinese women (RR for period effect, from 1.06 [95% CI 1.01, 1.11] to 0.85 [95% CI 0.82, 0.89]; RR for cohort effect, from 0.71 [95% CI 0.65, 0.78] to 0.51 [95% CI 0.26, 1.03]) during the study period and birth cohort. The LCM in Chinese population aged 65 to 79 and Australian women aged 75 to 79 increased. Smoking and particulate matter (PM) contributed most to LCM in China, while smoking and occupational carcinogens contributed most in Australia. Decreasing period and cohort risks for LCM attributable to smoking and PM were more remarkable in Australia than in China. The LCM attributable to occupational carcinogens was higher in Australia than in China, particularly for those aged 60 to 79. Vigorous tobacco and PM control, which brought a substantial decline in LCM in Australia, may help reduce LCM in China. Australia should highlight LC prevention among people with occupational exposure. Chinese aged ≥ 65 and Australian women aged ≥ 75 should be the priorities for LC interventions.

## Introduction

The World Health Organization (WHO) has launched the Global Non-communicable Diseases Action Plan to reduce premature mortality from non-communicable diseases by one-fourth by 2025^1^. The third United Nations Sustainable Development Goal also aims at reducing premature mortality from non-communicable diseases by one-third by 2030^2^. As the first leading cause of cancer deaths worldwide^3^, lung cancer (LC) is a major obstacle to achieving these goals.

The Western Pacific Region is one of the world’s most diverse regions and it plays an influential role in improving health outcomes on a global platform. The burden of LC mortality (LCM) in this region would greatly affect the achievement of global targets for non-communicable diseases control. Reducing the rising burden of LCM requires the coordinated efforts of all countries in this region.

China and Australia are the two largest countries in the Western Pacific Region, each with distinct social and environmental context. China has one-fifth of the world’s population and it is undergoing rapid industrialization and urbanization. Tobacco use in China remains at a high level, and it differed dramatically between men and women^4^. Australia, with relatively small population size, is one of the most highly-developed countries. Tobacco use in Australian men is much lower than that in Chinese men, but tobacco use in Australian women is higher than that in Chinese women^4^. A comparative analysis of LC trend and drivers between China and Australia is vital to planning LC interventions, fostering strong partnerships in health, and drawing lessons for countries in similar situations in the Western Pacific Region.

Existing studies on LCM in these two countries have been restricted to age-specific rates by time period^5–7^. The patterns of independent effect of age, time period, and birth cohort on LCM have not been discerned and compared so far. Mortality increases with age in line with biological mechanism, unrelated to the time period or birth cohort, which is referred to the age effect. Period effect refers to the variation of risk factors over a period of time that has equal impact on different ages and birth cohorts. Cohort effect stands for the change affecting individuals born in the same year, and this impact will persist throughout the life cycle of this generation^8,9^. Decomposing and comparing the independent age, time period, and cohort effects on the trend of LCM rates in China and Australia are crucial for intervention evaluation and timely adjustment of control strategies in the two countries.

The Global Burden of Disease Study (GBD) 2019 uses the most recent data available to reveal the burden of diseases, injuries, and risk factors in different countries and territories across the world^10,11^. The unified estimation method in the GBD makes the disease burden and trend in different countries comparable.

In this paper, we examined and compared LCM rates and their relations to age, time period, and birth cohort in China and Australia from 1990 to 2019 using data from the GBD 2019.

## Methods

### Study data

The GBD 2019 reported mortality by age and sex for 285 causes in 195 countries and territories from 1990 to 2019. We retrieved age-, sex-, and year-specific numbers of LC death, those attributed to a variety of risk factors, and sex-specific LC age-standardized mortality rates (ASMRs) in China and Australia using a GBD data extraction tool (http://ghdx.healthdata.org/gbd-results-tool) provided by the Global Health Data Exchange.

The full details of the GBD methodology had been reported elsewhere^3,10,11^. LCM were estimated based on data from vital registration, cancer registry, and verbal autopsy. Data sources in China included the Disease Surveillance Points, Maternal and Child Surveillance System, Chinese Center for Disease Control and Prevention Cause of Death Reporting System, and Cancer registry^6^. Data sources in Australia included the Australasian Association of Cancer Registries, Australian Institute of Health and Welfare, and Australia Vital Registration^12^. Cancer registry was used to compute mortality-to-incidence ratios (MIRs) that were inputted into a linear mixed model with covariates. Cancer mortality was then estimated by combining cancer incidence data with predicted MIRs. Both observed (from vital registration) and estimated cancer mortality (estimated by MIRs and incidence) were uploaded into a Cause of Death Ensemble model (CODEm). Estimated single causes of mortality were adjusted by the CoDCorrect algorithm to ensure that the sum of deaths from all single causes equal to the all-cause death estimation.

The GBD included risk-outcome pairs that meet the World Cancer Research Fund-defined criteria and it used the comparative risk assessment approach to evaluate the relationships between risk factors and outcomes^11^. Briefly, there were six steps in the estimation: (1) estimating relative risk through meta-analyses of published randomized controlled trials and cohort studies; (2) estimating the distribution of each risk factor by searching published studies, household surveys, census, administrative data, ground monitor data, and satellite remote sensing data and by Bayesian statistical models; (3) determining the level of exposure with minimum risk, i.e., the theoretical minimum risk exposure level; (4) computing age-sex-year specific population attributable fractions for each risk factor-outcome pair by embracing relative risk, risk factor’s distribution, and theoretical minimum risk exposure level (the population attributable fraction represented the proportion by which the outcome would be avoided if the exposure to a certain risk factor decreased to the counterfactual scenario of theoretical minimum risk exposure level); (5) estimating the uncertainty of population attributable fraction by taking into account all sources of uncertainty from the estimation mentioned above; and (6) computing the attributable deaths by multiplying the population attributable fraction by the total deaths. We included the five most important common risk factors (i.e., smoking, particulate matter (PM), occupational exposure to carcinogens, secondhand smoke, and low in fruits) for LC in both countries to highlight their contributions.

Age-, sex-, and year-specific population data of China and Australia were also collected from the GBD. These figures were estimated based on the data from censuses and population registry location-years in the respective country^13^.

### Statistical analysis

Number of LC deaths and population collected from the GBD were presented for each 5-year-old age group, each sex, and each year from 1990 to 2019. We extracted those aged between 20 and 79 years old and grouped them into 5-year periods, i.e., 1990–1994, 1995–1999, 2000–2004, 2005–2009, 2010–2014, and 2015–2019. Birth cohort was calculated by subtracting age of death from the time period of death. The data for analysis comprised 17 consecutive birth cohorts including those born between 1913–1917 and 1993–1997. The median of the study period, i.e., 2000–2004 was the reference period and the median of the birth cohort, i.e., 1953–1957 was the reference cohort. In terms of LCM attributable to risk factors, we only extracted those of people aged between 30 and 79 due to the small number of LC deaths associated with each risk factor under the age of 30. The reference cohort was 1948–1952 for the LCM attributable to risk factors.

We applied the age-period-cohort modeling to illustrate the effects of age, time period, and cohort on LCM using the numbers of LC deaths (counts) and corresponding person-years at risk (population) for specific age and sex groups and calendar time periods^8^. We estimated the rate ratios (RRs) of LCM in different periods (period effects) and cohorts (cohort effects) relative to the reference points specified above. We also computed the net drift, local drifts, cross-sectional age curve, and longitudinal age curve of LCM rate. Net drift represents the annual percentage change in age-adjusted LCM rate over time, which indicates the overall log-linear trend by period and cohort. Local drifts represent the annual percentage changes of LCM rate in each age group over time, which indicate the age-specific log-linear trends by period and cohort. Cross-sectional age curve represents the expected age-specific rates in reference period adjusted for cohort effect. Longitudinal age curve represents the expected age-specific rates in reference cohort adjusted for period effect.

We conducted the analyses through the age-period-cohort Web Tool from the National Cancer Institute that uses the statistical method of weighted least squares and the assumption that the count data follow a Poisson distribution^8^. To address the identifiability problem of the age-period-cohort model, the Web Tool expresses the model in terms of those estimable functions of the age, period, and cohort parameters following Holford’s Age-period-cohort model. In Holford’s Age-period-cohort model, each set of effects are presented by linear combinations of the curvature effects. Functions of the slopes in the age, period, and cohort effects are estimable^14^. The equations are as follows.

For basic age-period-cohort model,

$$\rho =\text{log}{(\theta }_{\mathit{apc}}/{N}_{\mathit{apc}})={\alpha }_{a}+{\pi }_{p}+{\gamma }_{c},$$

where $\rho$ represents the log transformation of expected LCM rate. $\alpha , \pi, \,\text{and}\, \gamma$ represent age, period, and cohort effects, respectively.

For longitudinal form,

$${\rho }_{ac}=\mu +\left({\alpha }_{L}+{\pi }_{L}\right)\left(a-\overline{a }\right)+\left({\pi }_{L}+{\gamma }_{L}\right)\left(c-\overline{c }\right)+{\stackrel{\sim }{\alpha }}_{a}+{\stackrel{\sim }{\pi }}_{p}+{\stackrel{\sim }{\gamma }}_{c},$$

where $({\alpha }_{L}+{\pi }_{L})$ is the longitudinal age trend, ${(\pi }_{L}+{\gamma }_{L})$ is the net drift, and ${\stackrel{\sim }{\alpha }}_{a}, {\stackrel{\sim }{\pi }}_{p},\text{and} {\stackrel{\sim }{\gamma }}_{c}$ are the deviations for age, period, and cohort, respectively.

For cross-sectional form,

$${\rho }_{ap}=\mu +\left({\alpha }_{L}-{\gamma }_{L}\right)\left(a-\overline{a }\right)+\left({\pi }_{L}+{\gamma }_{L}\right)\left(p-\overline{p }\right)+{\stackrel{\sim }{\alpha }}_{a}+{\stackrel{\sim }{\pi }}_{p}+{\stackrel{\sim }{\gamma }}_{c},$$

where $\left({\alpha }_{L}-{\gamma }_{L}\right)$ is the cross-sectional age trend, $({\pi }_{L}+{\gamma }_{L})$ is the net drift, and ${\stackrel{\sim }{\alpha }}_{a}, {\stackrel{\sim }{\pi }}_{p},\,\text{and}\, {\stackrel{\sim }{\gamma }}_{c}$ are the deviations for age, period, and cohort, respectively.

The Wald Chi-Square tests were employed to test the significance of the parameters. *P*-value of less than 0.05 (two-sides) was considered as statistically significant.

### Ethical statement

This study was the secondary analysis of the publicly available data from the GBD 2019. All the data used in this study were aggregated data without any identification information. All methods of this study were performed in accordance with the guidelines and regulations of the Declaration of Helsinki.

## Results

### Trends in LC ASMRs

Tables 1 and 2 show the changes in LC ASMR, LC death number, and relative proportion of LC death among all causes of death in China and Australia from 1990 to 2019 and those attributable to prominent risk factors. During this period, LC ASMR increased from 46.33 [95% uncertainty interval (UI): 38.78, 55.03] to 58.10 [95% UI: 46.53, 70.89] per 100,000 population among Chinese men, while it decreased from 54.66 [95% UI: 52.68, 56.51] to 30.13 [95% UI: 27.88, 32.31] per 100,000 population among Australian men. In Chinese men, the proportion of LC death in all causes of death increased from 3.88 to 8.39%, while in Australian men, the proportion decreased from 7.53 to 6.76%. Smoking was the most important risk factor for LC among men in both countries in 1990 and 2019. PM and occupational exposure to carcinogens were the second most important risk factors for LC in Chinese men and Australian men respectively. Among Chinese men, LC ASMR attributable to all risk factors increased except for diet low in fruits, and that related to smoking increased the most (from 37.27 [95% UI: 31.16, 44.35] to 47.66 [95% UI: 37.89, 58.45] per 100,000 population). By contrast, LC ASMR attributable to all risk factors decreased among Australian men, and that related to smoking decreased the most (from 42.31 [95% UI: 40.47, 44.08] to 17.62 [95% UI: 16.00, 19.20] per 100,000 population) (Table 1).

**Table 1:** Lung cancer mortality among men in China and Australia between 1990 and 2019. †ASMR: age-standardized mortality rate; ‡PM: particulate matter including ambient particulate matter pollution and household air pollution from solid fuels.

	China		Australia	
	1990	2019	1990	2019
**Lung cancer mortality**				
ASMR, per 100,000^†^	46.33 (38.78, 55.03)	58.10 (46.53, 70.89)	54.66 (52.68, 56.51)	30.13 (27.88, 32.31)
Deaths, × 1000	177.93 (145.87, 214.39)	523.19 (413.19, 647.41)	4.79 (4.62, 4.94)	6.01 (5.54, 6.45)
Relative proportion in all causes of death, %	3.88	8.39	7.53	6.76
**Lung cancer mortality attributable to smoking**				
ASMR, per 100,000^†^	37.27 (31.16, 44.35)	47.66 (37.89, 58.45)	42.31 (40.47, 44.08)	17.62 (16.00, 19.20)
Deaths, × 1000	140.17 (114.73, 168.90)	430.46 (339.24, 533.89)	3.76 (3.60, 3.91)	3.51 (3.18, 3.83)
Relative proportion in lung cancer deaths, %	78.78	82.28	78.46	58.36
**Lung cancer mortality attributable to PM**^‡^				
ASMR, per 100,000^†^	15.16 (10.80, 19.99)	15.86 (11.37, 21.28)	2.33 (0.24, 5.79)	0.93 (0.23, 1.82)
Deaths, × 1000	58.19 (41.18, 77.99)	143.13 (101.89, 195.14)	0.20 (0.02, 0.51)	0.19 (0.05, 0.36)
Relative proportion in lung cancer deaths, %	32.69	27.37	4.27	3.10
**Lung cancer mortality attributable to occupational carcinogens**				
ASMR, per 100,000^†^	3.65 (2.53, 5.14)	5.10 (3.43, 7.11)	26.38 (19.93, 32.19)	14.09 (10.56, 17.44)
Deaths, × 1000	14.64 (9.87, 20.54)	45.69 (30.54, 64.90)	2.30 (1.75, 2.81)	2.90 (2.18, 3.58)
Relative proportion in lung cancer deaths, %	8.23	8.73	48.11	48.20
**Lung cancer mortality attributable to secondhand smoke**				
ASMR, per 100,000^†^	2.81 (1.64, 4.43)	3.59 (2.05, 5.57)	2.52 (1.47, 3.79)	0.95 (0.54, 1.48)
Deaths, × 1000	10.53 (6.18, 16.48)	32.09 (18.31, 50.18)	0.22 (0.13, 0.33)	0.18 (0.10, 0.29)
Relative proportion in lung cancer deaths, %	5.92	6.13	4.66	3.07
**Lung cancer mortality attributable to diet low in fruits**				
ASMR, per 100,000^†^	2.43 (0.81, 3.80)	2.14 (0.55, 3.53)	2.48 (0.68, 3.70)	1.26 (0.31, 1.91)
Deaths, × 1000	9.32 (3.19, 14.83)	18.98 (5.00, 31.52)	0.22 (0.06, 0.32)	0.25 (0.06, 0.38)
Relative proportion in lung cancer deaths, %	5.25	3.63	4.53	4.17

**Table 2:** Lung cancer mortality among women in China and Australia between 1990 and 2019. †ASMR: age-standardized mortality rate; ‡PM: particulate matter including ambient particulate matter pollution and household air pollution from solid fuels.

	China		Australia	
	1990	2019	1990	2019
**Lung cancer mortality**				
ASMR, per 100,000^†^	18.63 (15.62, 21.64)	22.86 (18.52, 27.52)	16.38 (15.52, 17.06)	17.80 (15.93, 19.34)
Deaths, × 1000	78.40 (65.03, 91.67)	233.98 (189.18, 282.75)	1.77 (1.67, 1.84)	4.02 (3.56, 4.40)
Relative proportion in all causes of death, %	2.08	5.28	3.27	4.91
**Lung cancer mortality attributable to smoking**				
ASMR, per 100,000^†^	3.64 (2.77, 4.63)	6.08 (4.84, 7.53)	11.20 (10.47, 11.91)	9.57 (8.47, 10.59)
Deaths, × 1000	14.18 (10.66, 18.28)	61.26 (48.72, 76.49)	1.21 (1.13, 1.29)	2.14 (1.88, 2.38)
Relative proportion in lung cancer deaths, %	18.10	26.18	68.76	53.28
**Lung cancer mortality attributable to PM**^‡^				
ASMR, per 100,000^†^	6.25 (4.53, 8.13)	6.39 (4.61, 8.45)	0.72 (0.10, 1.74)	0.55 (0.14, 1.10)
Deaths, × 1000	26.25 (18.94, 34.30)	65.46 (47.13, 86.52)	0.08 (0.01, 0.19)	0.13 (0.03, 0.25)
Relative proportion in lung cancer deaths, %	33.48	27.98	4.39	3.12
**Lung cancer mortality attributable to occupational carcinogens**				
ASMR, per 100,000^†^	1.36 (0.89, 1.90)	1.63 (1.07, 2.27)	2.19 (1.43, 3.54)	2.79 (1.76, 3.87)
Deaths, × 1000	5.94 (3.87, 8.21)	17.17 (11.25, 24.07)	0.25 (0.16, 0.40)	0.67 (0.42, 0.94)
Relative proportion in lung cancer deaths, %	7.56	7.33	14.00	16.62
**Lung cancer mortality attributable to secondhand smoke**				
ASMR, per 100,000^†^	2.30 (1.35, 3.45)	2.62 (1.55, 3.99)	0.66 (0.39, 1.02)	0.51 (0.29, 0.78)
Deaths, × 1000	9.81 (5.73, 14.77)	27.07 (16.04, 41.23)	0.07 (0.04, 0.10)	0.11 (0.06, 0.16)
Relative proportion in lung cancer deaths, %	12.50	11.56	3.81	2.64
**Lung cancer mortality attributable to diet low in fruits**				
ASMR, per 100,000^†^	0.99 (0.33, 1.54)	0.81 (0.22, 1.26)	0.76 (0.22, 1.14)	0.76 (0.20, 1.17)
Deaths, × 1000	4.16 (1.40, 6.49)	8.21 (2.22, 12.87)	0.08 (0.02, 0.12)	0.17 (0.05, 0.27)
Relative proportion in lung cancer deaths, %	5.31	3.51	4.67	4.27

From 1990 to 2019, there was an increase in LC ASMR in both Chinese women (from 18.63 [95% UI: 15.62, 21.64] to 22.86 [95% UI: 18.52, 27.52] per 100,000 population) and Australian women (from 16.38 [95% UI: 15.52, 17.06] to 17.80 [95% UI: 15.93, 19.34] per 100,000 population). Proportions of LC death in all causes of death also increased among women in both countries (Chinese women, from 2.08 to 5.28%; Australian women, from 3.27 to 4.91%) during this period. In Chinese women, PM and smoking were the two most important risk factors in 2019, accounting for 27.98% and 26.18% of total LC deaths, respectively. In Australian women, smoking was the leading risk factor, followed by occupational exposure to carcinogens. They were associated with 53.28% and 16.62% of total LC deaths in 2019, respectively. LC ASMR attributable to smoking was consistently higher in Australian women than in their Chinese counterparts, whereas LC ASMR attributable to PM was substantially lower in Australian women than in their Chinese counterparts. ASMR attributable to all risk factors increased among Chinese women except for diet low in fruits. Conversely, ASMR attributable to all risk factors decreased among Australian women except for occupational carcinogens (from 2.19 [95% UI: 1.43, 3.54] to 2.79 [95% UI: 1.76, 3.87] per 100,000 population) (Table 2).

**Figure 1:**
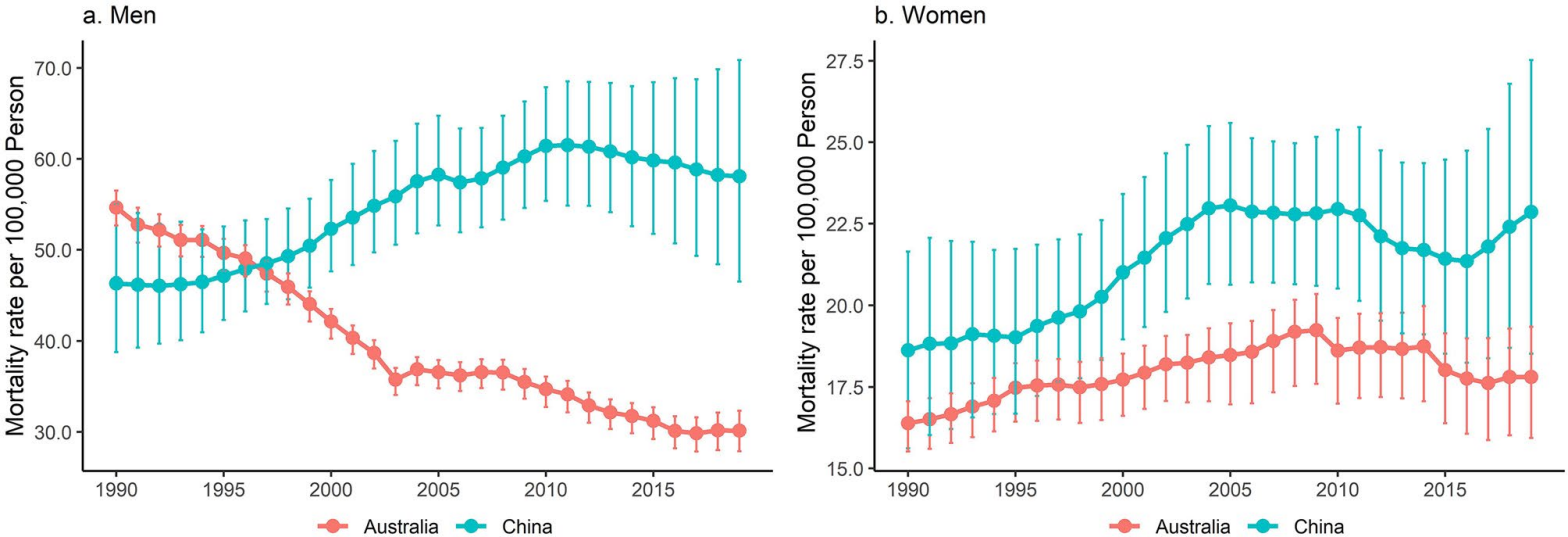
Age-standardized mortality rate of lung cancer by sex in China and Australia, 1990 to 2019.

Annual data show that LC ASMR in Chinese men generally increased from 1990 to 2011 and decreased after 2012. LC ASMR in Australian men constantly declined during the study period. LC ASMR in Chinese women declined slightly after 2005 but started to increase since 2017. LC ASMR in Australian women increased initially and then declined after 2010 (Fig. 1).

### Trends in age- and cohort-specific LCM rates

**Figure 2:**
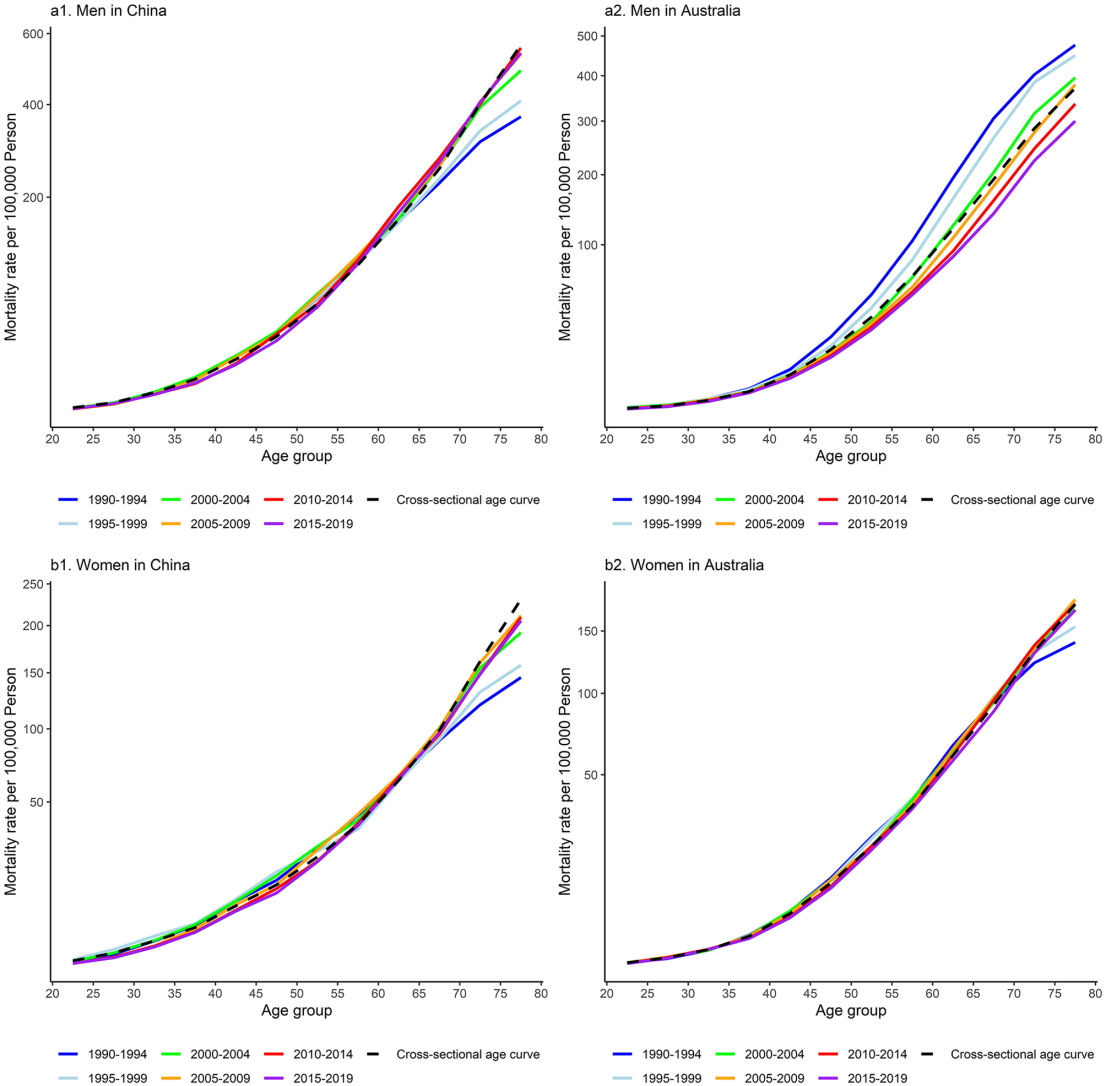
Age-specific mortality rates of lung cancer by period and sex in China and Australia, 1990 to 2019. The study period was organized into 5-year periods from 1990–1994, 1995–1999, 2000–2004, 2005–2009, 2010–2014, to 2015–2019. (**a1,b1**) Reveal that lung cancer mortality rate increased among men and women aged 65 or older in China over the study period (P < 0.001). (**a2**) Shows that in Australian men, lung cancer mortality rate decreased over the study period in all age groups (P < 0.001). (**b2**) Shows that lung cancer mortality rate increased among Australian women aged 70 or older (P < 0.001). Cross-sectional age curves present the expected age-specific rates in reference period, i.e., 2000 to 2004, after adjusting for cohort effects.

Figure 2 shows that LCM rate increased with age. Figure 2a1,b1 reveal that LCM rate increased among men and women aged 65 or older in China over the study period (*P* < 0.001). Figure 2a2 shows that in Australian men, LCM rate decreased over the study period in all age groups (*P* < 0.001). Figure 2b2 shows that there was an upward trend in LCM rate among Australian women aged 70 or older (*P* < 0.001). Cross-sectional age curves present the expected age-specific rates in reference period, i.e., 2000 to 2004, after adjusting for cohort effects among both genders in the two countries.

**Figure 3:**
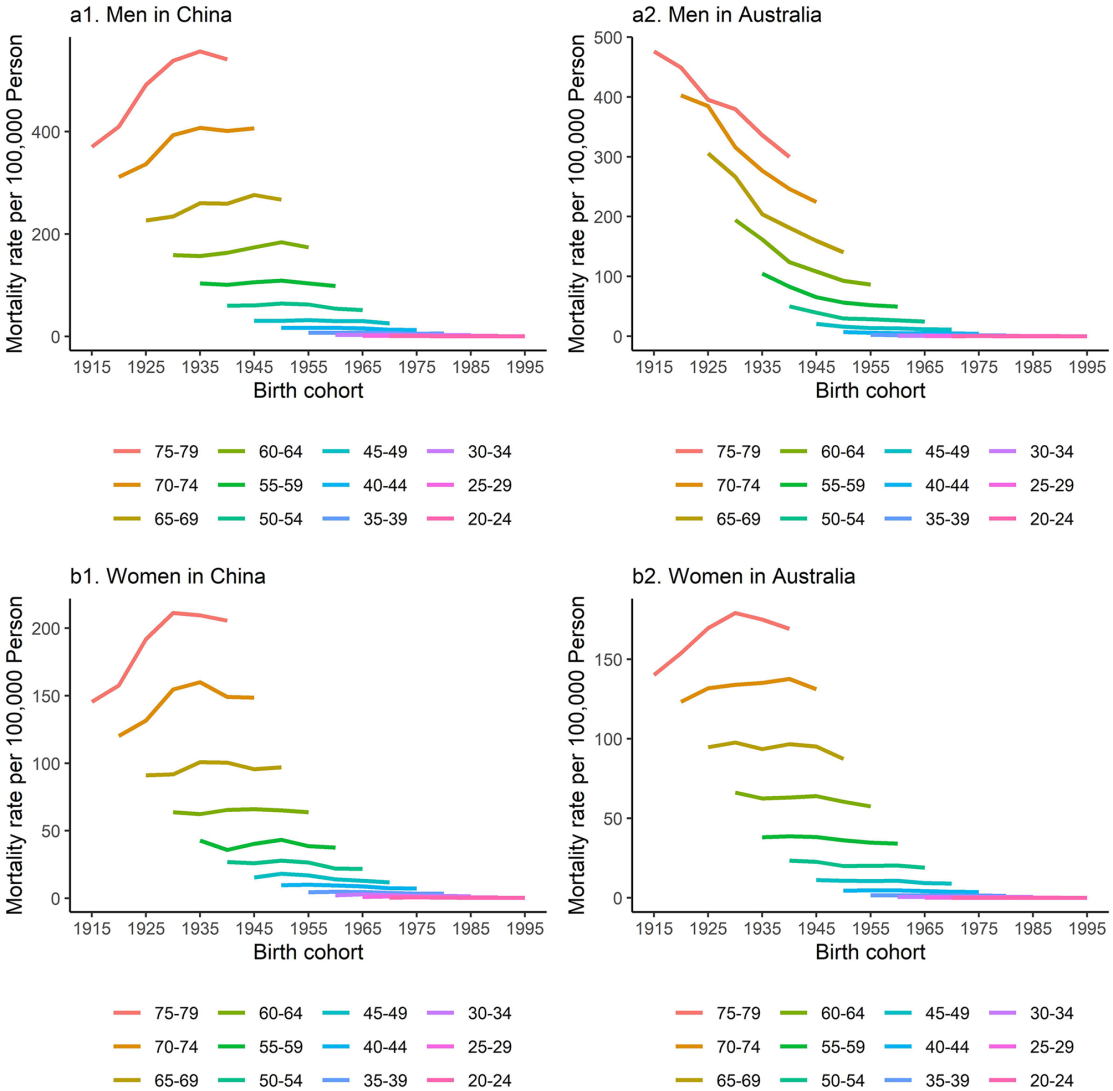
Cohort-specific mortality rates of lung cancer by age and sex in China and Australia, 1990 to 2019. Data of lung cancer mortality were grouped into 17 successive birth cohorts which include those born between 1913–1917 and 1993–1997, and 12 age groups between 20–24 and 75–79. (**a1**) Shows that lung cancer mortality rate increased over birth cohorts among Chinese men aged between 60 and 79, indicating a higher rate in more recent birth cohorts among these age groups. On the contrary, there was a decreasing trend over birth cohorts among Chinese men aged between 20 and 59, indicating a lower mortality rate in more recent birth cohorts in these relatively young age groups. (**a2**) Shows that lung cancer mortality rates decreased over birth cohorts in all age groups of Australian men. This suggests a lower risk of lung cancer death in more recent birth cohorts across all ages. (**b1**) Shows that the trend of age-specific lung cancer mortality rates with birth cohort in Chinese women was similar to that in Chinese men. (**b2**) Shows that Australian women aged between 70 and 79 have experienced an increasing trend over birth cohorts, while those aged between 20 and 69 have experienced a decreasing trend over birth cohorts (P < 0.001 for all).

Figure 3 shows cohort-specific LCM rates by age and sex in China and Australia. Among Chinese men, LCM rate presented an increasing trend over birth cohorts among those aged between 60 and 79, indicating a higher mortality rate in more recent birth cohorts among these age groups. On the contrary, there was a decreasing trend over birth cohorts among Chinese men aged between 20 and 59, indicating a lower mortality rate in more recent birth cohorts in these relatively young age groups (Fig. 3a1). Among Australian men, LCM rates showed a decreasing trend over birth cohorts in all age groups. This suggests a lower risk of LC death in more recent birth cohorts across all ages (Fig. 3a2). The trend of age-specific LCM rates with birth cohort in Chinese women was similar to that in Chinese men (Fig. 3b1). In Australia, women aged between 70 and 79 have experienced an increasing trend over birth cohorts, while those aged between 20 and 69 have experienced a decreasing trend over birth cohorts (Fig. 3b2) (*P* < 0.001 for all).

### Net drift and local drift values for LCM rates

**Figure 4:**
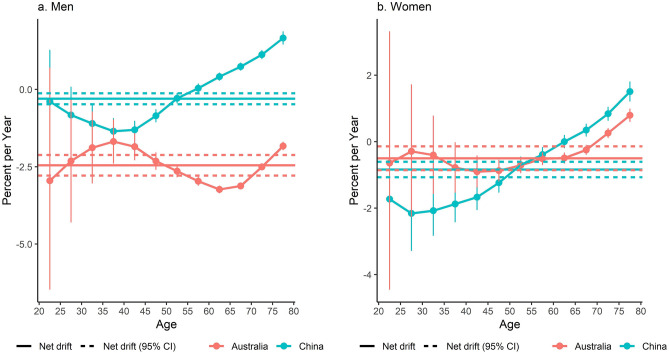
Net drift and local drift values for lung cancer mortality rate by sex in China and Australia, 1990 to 2019. Net drift represents the annual percentage change in age-adjusted lung cancer mortality rate over time, which indicates the overall log-linear trend by period and cohort. Local drifts represent the annual percentage changes of lung cancer mortality rate in each age group over time, which indicate the age-specific log-linear trends by period and cohort. (**a**) Shows that the net drift values were negative among both genders in the two countries, with Australian men having a significantly lower value than Chinese men. This indicates that the overall lung cancer mortality rate decreased more strikingly in Australian men than their Chinese counterparts. (**b**) Shows that the difference in the net drift value between Chinese women and Australian women was nonsignificant. The local drift values were positive in Chinese men and women aged between 65 and 79, suggesting lung cancer mortality rates were increasing in these age groups. In Australia, lung cancer mortality rates were decreasing with the exception of women aged between 75 and 79 (local drift values were positive in women of these age groups).

Figure 4 reports net and local drift values for LCM rates by sex in China and Australia. The net drift values were negative among both genders in the two countries, with Australian men (− 2.45% [95% CI − 2.79, − 2.12]) having a significantly lower value than Chinese men (− 0.30% [95% CI − 0.48, − 0.12]). This indicates that the overall LCM rate decreased more strikingly in Australian men than their Chinese counterparts. Among women, the difference in the net drift value between China and Australia was nonsignificant (Chinese women (− 0.84% [95% CI − 1.07, − 0.60]); Australian women (− 0.50% [95% CI − 0.86, − 0.14])).

The local drift values were positive in Chinese men and women aged between 65 and 79, suggesting LCM rates were increasing in these age groups. In Australia, LCM rates were decreasing with the exception of women aged between 75 and 79 (local drift values were positive in women of these age groups).

### Age, period, and cohort effects on LCM

**Figure 5:**
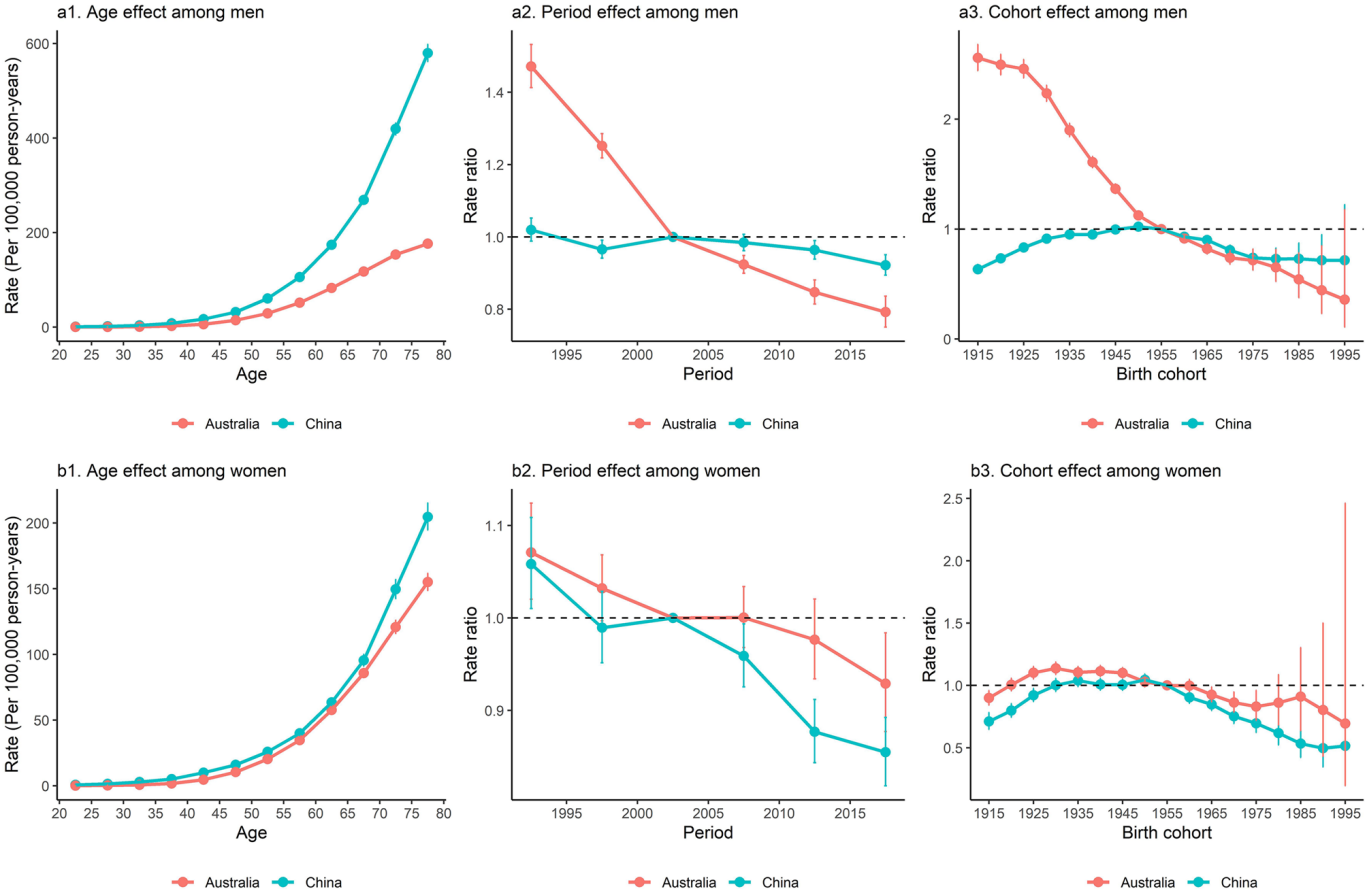
Parameter estimates of age, period, and cohort effects on lung cancer mortality in China and Australia, 1990 to 2019. (**a1,b1**) show the longitudinal age curves of lung cancer mortality (per 100,000 person-years) and the 95% CIs among men and women in the two countries, respectively, representing the expected age-specific rates in reference cohort adjusted for period effect. (**a2,b2**) show the rate ratios of lung cancer mortality in different periods (period effects) relative to the reference period (2000–2004) adjusted for age and cohort effect, and the 95% CIs among men and women in the two countries, respectively. (**a3,b3**) show the rate ratios of lung cancer mortality in different cohorts (cohort effects) relative to the reference cohort (1953–1957) adjusted for age and period effect, and the 95% CI among men and women in the two countries, respectively.

Age effect showed an exponential distribution among men in both countries, with China having a higher LCM rate than Australia across all age groups (Fig. 5a1). Period effect on LCM decreased dramatically among Australian men (RR from 1.47 [95% CI 1.41, 1.53] to 0.79 [95% CI 0.75, 0.84] during the entire study period), while that among Chinese men was almost unchanged (RR from 1.02 [95% CI 0.99, 1.05] to 0.92 [95% CI 0.89, 0.95] during the entire study period) (Fig. 5a2). Cohort effect constantly declined among Australian men (RR from 2.56 [95% CI 2.44, 2.68] to 0.36 [95% CI 0.11, 1.18] during the entire study cohort), while it presented an increasing trend among Chinese men born before 1955 and a decreasing trend after that (RR from 0.63 [95% CI 0.59, 0.68] to 1.00 between birth cohort of 1915 and 1955, and from 1.00 to 0.72 [95% CI 0.42, 1.22] between birth cohort of 1955 and 1995) (Fig. 5a3).

Chinese women had higher LCM rates than Australian women across all age groups (Fig. 5b1). Period effect on LCM declined more steeply among Chinese women than in Australian women (RR from 1.06 [95% CI 1.01, 1.11] to 0.85 [95% CI 0.82, 0.89] in Chinese women, and from 1.07 [95% CI 1.02, 1.12] to 0.93 [95% CI 0.88, 0.98] in Australian women) (Fig. 5b2). The decline of cohort effect on LCM in Australian women started at around 1935 (RR from 1.10 [95% CI 1.06, 1.15] to 0.69 [95% CI 0.20, 2.46] during the birth cohort of 1935 and 1995) and the decline in Chinese women started at around 1955 (RR from 1.00 to 0.51 [95% CI 0.26, 1.03] during the birth cohort of 1955 and 1995) (Fig. 5b3).

### Age, period, and cohort effects on LCM attributable to risk factors

Supplementary Figures S1–S5 presents the age-period-cohort effect on LCM attributable to each risk factor. Chinese men had higher LCM rates attributable to smoking than Australian men across all age groups. LCM rate attributable to smoking among Australian men declined rapidly over the study period (RR from 1.53 [95% CI 1.47, 1.60] to 0.68 [95% CI 0.65, 0.2]), whereas that among Chinese men barely changed (RR from 0.98 [95% CI 0.95, 1.02] to 0.93 [95% CI 0.90, 0.96]). The cohort effect showed that Chinese men born after 1955 had a favorable trend of LCM rate associated with smoking (RR from 0.98 [95% CI 0.96, 1.01] to 0.69 [95% CI 0.43, 1.08] between the birth cohort of 1955 and 1985), while the rate associated with smoking among Australian men continued improving over the birth cohort (RR from 3.10 [95% CI 2.94, 3.25] to 0.34 [95% CI 0.12, 0.95] during the entire study cohort)**.** LCM rate attributable to smoking was consistently higher among Australian women than that among Chinese women across all age groups. LCM rate attributable to smoking constantly declined among Australian women over the study period (RR from 1.15 [95% CI 1.09, 1.21] to 0.83 [95% CI 0.78, 0.88]), while that among Chinese women showed a fluctuating trend (RR from 0.78 [95% CI 0.73, 0.84] to 0.88 [95% CI 0.81, 0.94]). The cohort effect on LCM attributable to smoking declined among Australian women but generally increased among Chinese women (RR from 0.60 [95% CI 0.54, 0.66] to 1.04 [95% CI 0.26, 4.14] in Chinese women, and from 1.23 [95% CI 1.13, 1.32] to 0.56 [95% CI 0.19, 1.63] in Australian women) (Supplementary Fig. S1).

LCM rates attributable to PM, secondhand smoke, and diet low in fruits were all significantly higher among Chinese men and women than their Australian counterparts across all ages, while LCM rate attributable to occupational carcinogens was significantly higher among Australian population than that in Chinese population at the age between 60 and 79. The period and cohort effects on the LCM attributable to PM, occupational carcinogens, secondhand smoke, and diet low in fruits declined more steeply in Australian men than in Chinese men, while the period and cohort effects on the LCM attributable to occupational carcinogens and diet low in fruits declined more steeply in Chinese women than in Australian women (Supplementary Figs. S2–S5). Supplementary Figures S6 and S7 further decompose the exposure to PM into ambient PM and household air pollution from solid fuels. The decline in the period and cohort effects on LCM attributable to PM in China was mainly due to those attributable to household air pollution from solid fuels, and those attributable to ambient PM were still on the rise.

## Discussion

The trends of LCM rate differed widely between China and Australia. Chinese men and women, particularly men, had markedly higher LC ASMR than their Australian counterparts in 2019. From 1990 to 2019, the period effects on LCM were more favorable in Australian men and Chinese women. The cohort effect on LCM constantly declined in Australian men, while such a decline in Australian women, and Chinese men and women started in much later years. Smoking was the most important risk factor associated with LCM in both countries, and PM and occupational carcinogens were the second most important risk factors for LCM in China and Australia, respectively.

Smoking was the leading risk factor associated with LCM among Chinese men, Australian men and women. The difference in smoking prevalence could mainly explain the striking differences in LCM rate and trend between the two countries. To reduce smoking prevalence and related detrimental health effects, Australia imposed strict tobacco control legislations and measures including smoke-free laws in all states and territories, tax hike on tobacco products, tobacco plain packaging, health warnings on tobacco products, tobacco advertising bans, and e-cigarette laws^15,16^. These unremitting efforts have brought a substantial and continuous decline in smoking prevalence in Australia. Previous studies reported that overall smoking prevalence rate in Australia had dropped to 12.8% in 2017, which was a 43.4% decline since 2001^17^. Data of 1980s and 1990s also suggested a striking decline in smoking prevalence (from 35.0 to 26.0% between 1980 and 1998)^18^. The benefit of such decrease in smoking prevalence has been demonstrated by the steep decline, particularly among Australian men, in the period and cohort effects on LCM attributable to smoking in our study results. The less favorable period trend in LCM for Australian women compared with men may result from the late beginning of decline in smoking prevalence among women^18^. A more pronounced decrease in LCM trend among Australian women is expected over the next 10 or 20 years.

Although China has signed up to the WHO Framework Convention on Tobacco Control and enforced tobacco advertising bans, it has no nationwide smoke-free legislation that prohibits tobacco smoking in public places or workplaces. Neither has it implemented strategies of tobacco taxes or plain packaging with graphic warnings^5,19^. Between 1996 and 2002, smoking prevalence rates of men and women in China had decreased only by 6.76% and 1.05% respectively^20^. The rate remained consistently high in Chinese men between 2003 and 2013 (47.2% in 2013)^19^. Because of traditional culture, Chinese women used to have a low smoking prevalence (2.7% in 2013), but there is report that the prevalence in young women had increased between 2003 and 2013^21^. Our study found that the period and cohort effects on LCM attributable to smoking among Chinese men and women remained stable over the study period. Comparing with Australia, China still needs to adopt more effective tobacco control measures in order to reduce the LCM burden attributable to smoking in the country.

LCM rates attributable to PM among both genders in China were substantially higher than their counterparts in Australia during the study period. In terms of trend development, period and cohort effects on LCM attributable to PM in Australia were decreasing more markedly than those in China. The decline in China was mainly due to that attributable to household air pollution from solid fuels, but that attributable to ambient PM were still on the rise. Rapid industrialization and urbanization have resulted in environmental pollution in the country. Fortunately, the Chinese government is aware of the serious health hazard of ambient PM and has taken actions to combat air pollution. The Action Plan of Air Pollution Prevention and Control, a national environmental protection policy, was enforced in 2013. It stipulated air quality targets for each prefecture-level city and promulgated detailed control measures (such as enforcing environmental protection laws, restricting motor vehicles, and updating household energy)^22^. It is encouraging that ambient PM_2.5_ concentration has declined in China in recent years (average annual concentration decreased from 67.4 in 2013 to 45.5 μg/m^3^ in 2017)^23,24^. LCM rate attributable to ambient PM will decline in the next decades if more stringent and sustained air pollution control measures are imposed in the country.

The level of ambient PM concentration in Australia (average daily concentration of PM_2.5_: 12.1–21.7 μg/m^3^, average daily concentration of PM_10_: 4.6–8.7 μg/m^3^) is considerably lower than in China and it stays within the WHO daily guideline values most of the time^25^. Although energy consumption and the number of vehicles have increased over the last two decades, ambient PM concentrations had declined slightly. This can be partly explained by the enforcement of emission reduction policies for vehicle exhaust and power generation in Australia^25^. Given the magnitude of urbanization and climate change in Australia, it is unlikely to further reduce ambient PM concentrations in this country.

Long-term exposure to carcinogens in the workplace was associated with 9% to 15% of LC^26^. The most important LC-related occupational carcinogen is asbestos^27^. LCM rate attributable to occupational carcinogens among Australian population over 60 years old was higher than that of Chinese counterparts of the same age, although LCM rate attributable to occupational carcinogens presented a downward trend among Australian men. Australia has completely banned the use of asbestos in 2003 but its impact on LC still exists. It is reported that the peak of the number of deaths due to malignancies related to asbestos occurred around 2020 in Australia^28^. More attention should be paid to early detection of LC for the elderly who have been exposed to occupational carcinogens in Australia. Health education needs to be strengthened to raise public awareness of identifying early symptoms and seeking medical diagnosis and treatment^29^.

LCM rate attributable to occupational carcinogens among Chinese population under the age of 60 was higher than that of the same age group in Australia, suggesting that LCM associated with occupational carcinogens cannot be ignored in China under rapid industrialization. The Chinese government has enacted national regulations for asbestos usage under safe conditions. However, the continued use of certain types of asbestos and the lack of health education on occupational carcinogen may further increase LCM burden attributable to occupational exposure. LC surveillance and health promotion for the protection against carcinogen are vitally needed in the workplace in China^30,31^.

Our study found that the increases of LCM rate in Chinese men and women occurred primarily among older people (65 to 79 years old), and the increase of LCM rate in Australian women occurred predominately among those aged 75 to 79 years old. These population should be the priorities for LC screening, early detection, and treatment.

This is the first study to compare age, period, and cohort effects on LCM and trends between China and Australia. The GBD Study 2019 collected all available data and used a unified method to estimate three-dimensional distributions of diseases and risk factors, and to facilitate comparison among countries. Our findings enable the health authorities to better understand the disparity in LCM burden between the two countries and to design more effective LC intervention strategies by learning from successful experience of the others. Another strength of this study is the application of age-period-cohort model that discerns three types of time-varying phenomena: age effect, period effect, and cohort effect on LCM attributable to several primary risk factors. The analyses provide a refined estimation crucial for identifying the priority intervention group and adjusting intervention measures.

Our study has several limitations. First, analyses of LCM over 80 years old and LCM attributable to risk factors under 30 years old were omitted from the study due to the small number of LC deaths in these age groups. Second, for the absence of data, we could not distinguish the different patterns of age, period, and cohort in LCM by histological type or stage. Adenocarcinoma and squamous cell carcinoma may be particularly associated with different risk factors. The contribution of smoking to squamous cell carcinoma is higher than that to adenocarcinoma^32,33^. Previous study reveals that compare to Australian women, Chinese women were more likely to have adenocarcinoma, indicating other risk factors may play an important role in LC of Chinese women^34^. Third, the estimates of LC deaths attributable to risk factors were based on the distribution of risk factors and relative risks. Some risk factors in the GBD such as exposure to occupational carcinogens were collected by less reliable mode (self-report). Moreover, the GBD assumed that relative risks were universal across all locations and time periods in spite of the possible variation among different subgroups. To improve the estimation, the GBD 2019 implemented explicit corrections for the distribution of risk factors. However, information bias might not be fully ruled out. As more data and evidence accumulate, future studies are needed to incorporate more precise data and take into account the disparity in relative risks among subgroups.

In conclusion, LCM rates and trends differed strikingly between China and Australia. Australia has seen a significant reduction in LCM rate, particularly for those attributable to smoking. More stringent control strategies for tobacco use and ambient PM would ease the increasingly heavy LCM burden in China and other low- and middle-income countries with similar challenges in the Western Pacific Region. Australia needs to pay more attention to the early detection of LC among elderly people with a history of occupational carcinogen exposure. People aged 65 to 79 in China and women aged 75 to 79 in Australia should be the priority for LC screening, early detection and treatment.

## Supplementary Information

Supplementary Figures.

## Data Availability

All data in this study are available at http://ghdx.healthdata.org/gbd-results-tool.
