# Supplementary material for: Age-period-cohort analysis of lung cancer mortality in China and Australia from 1990 to 2019
Source: Sci Rep. 2022 May 19;12:8410. doi: 10.1038/s41598-022-12483-z (PMC9120450; doi:10.1038/s41598-022-12483-z)

Supplementary Information

Age-period-cohort analysis of lung cancer mortality in China and Australia from 1990 to 2019

Ning Wang, PhD; Zhiwei Xu, PhD; Chi-Wai Lui, PhD; Baohua Wang, MD; Wenbiao Hu, PhD; Jing Wu, PhD

Supplementary Figure S1. Parameter estimates of age, period, and cohort effects on lung cancer mortality attributable to smoking in China and Australia, 1990 to 2019 2

Supplementary Figure S2. Parameter estimates of age, period, and cohort effects on lung cancer mortality attributable to particulate matter in China and Australia, 1990 to 2019 3

Supplementary Figure S3. Parameter estimates of age, period, and cohort effects on lung cancer mortality attributable to occupational carcinogens in China and Australia, 1990 to 2019 4

Supplementary Figure S4. Parameter estimates of age, period, and cohort effects on lung cancer mortality attributable to secondhand smoke in China and Australia, 1990 to 2019 5

Supplementary Figure S5. Parameter estimates of age, period, and cohort effects on lung cancer mortality attributable to diet low in fruits in China and Australia, 1990 to 2019 6

Supplementary Figure S6. Parameter estimates of age, period, and cohort effects on lung cancer mortality attributable to ambient particulate matter in China and Australia, 1990 to 2019 7

Supplementary Figure S7. Parameter estimates of age, period, and cohort effects on lung cancer mortality attributable to household air pollution from solid fuels in China and Australia, 1990 to 2019 8

Supplementary Figure S1. Parameter estimates of age, period, and cohort effects on lung cancer mortality attributable to smoking in China and Australia, 1990 to 2019

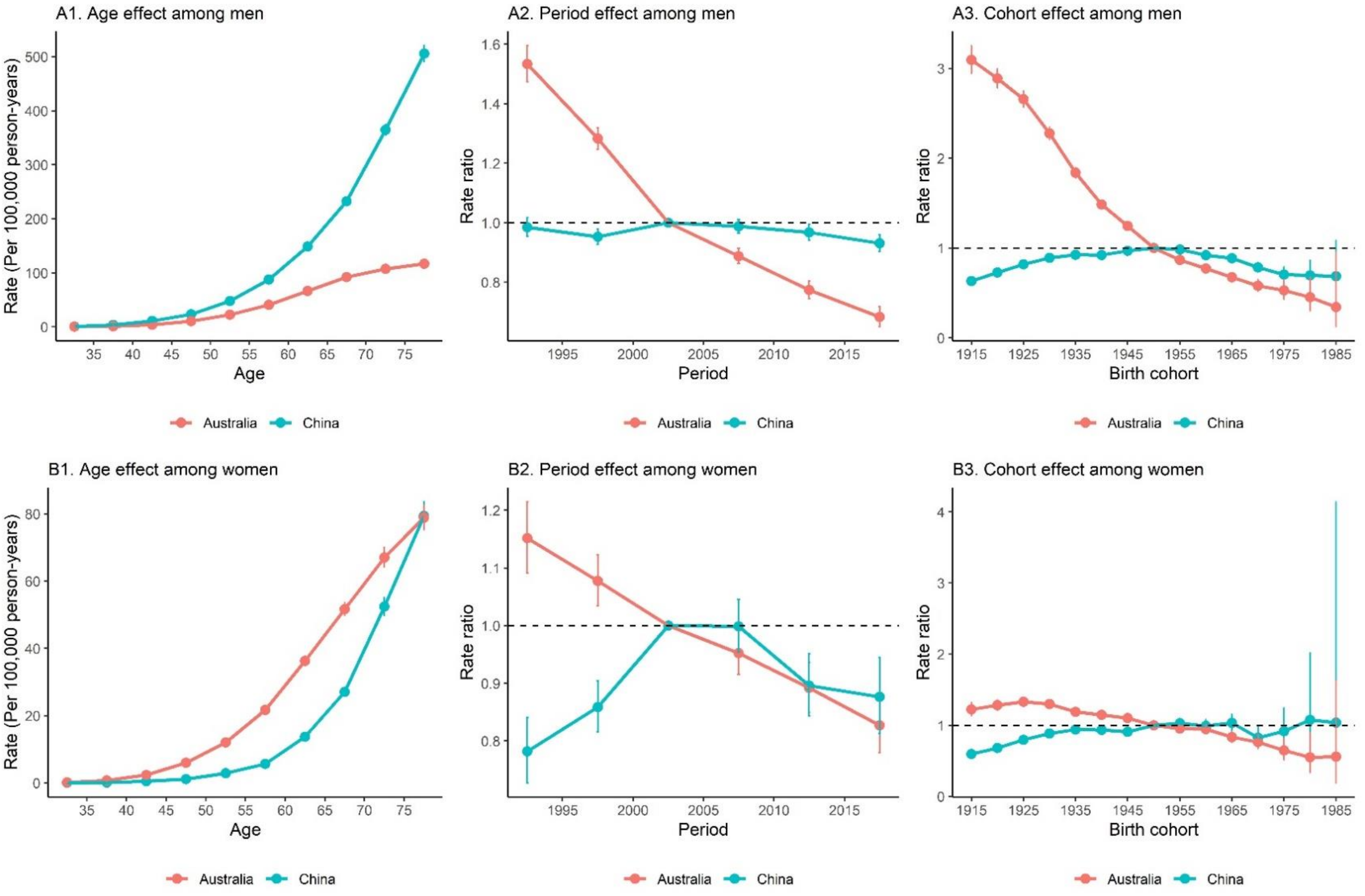

Supplementary Figure S2. Parameter estimates of age, period, and cohort effects on lung cancer mortality attributable to particulate matter in China and Australia, 1990 to 2019

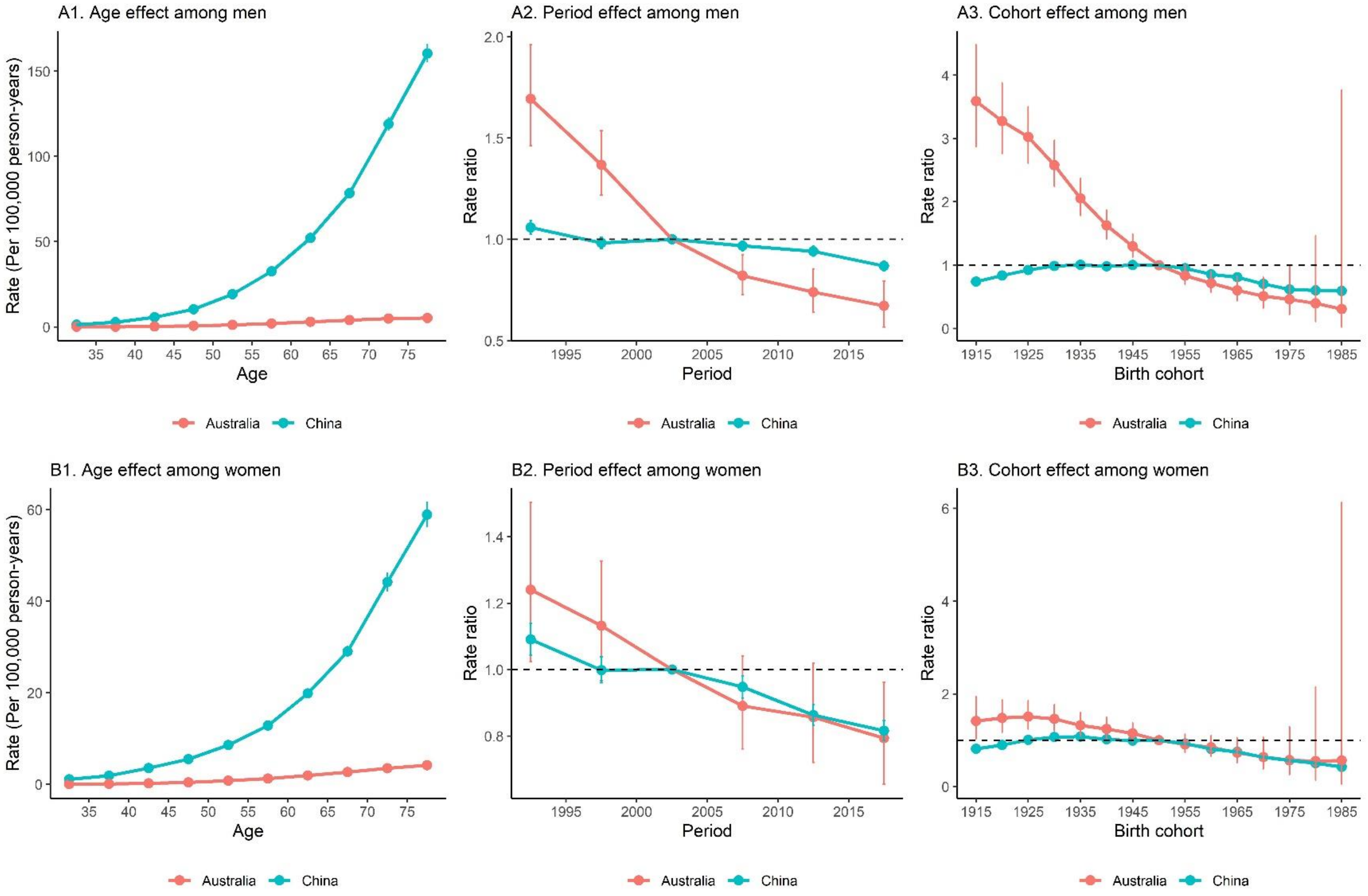

Supplementary Figure S3. Parameter estimates of age, period, and cohort effects on lung cancer mortality attributable to occupational carcinogens in China and Australia, 1990 to 2019

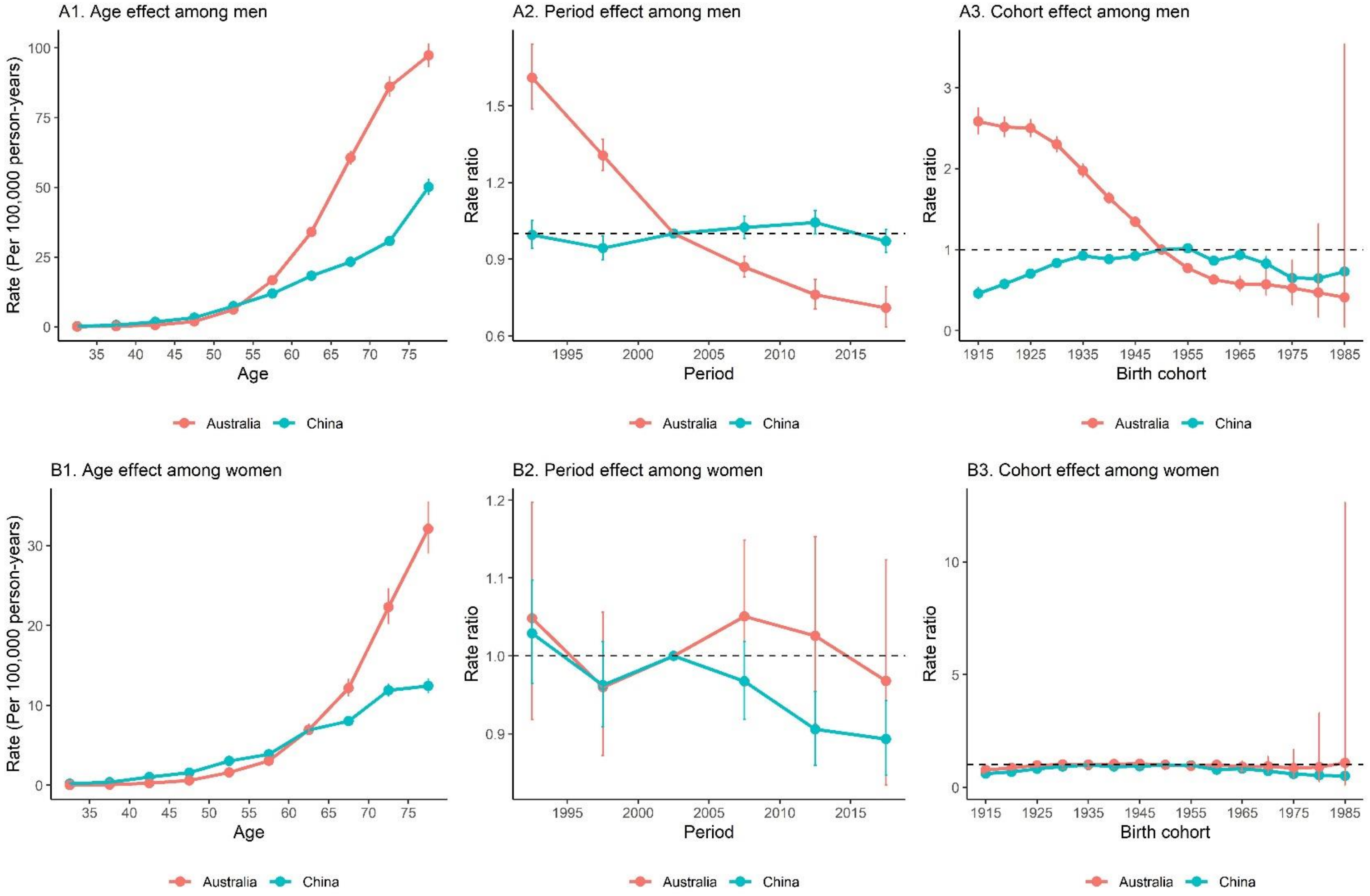

Supplementary Figure S4. Parameter estimates of age, period, and cohort effects on lung cancer mortality attributable to secondhand smoke in China and Australia, 1990 to 2019

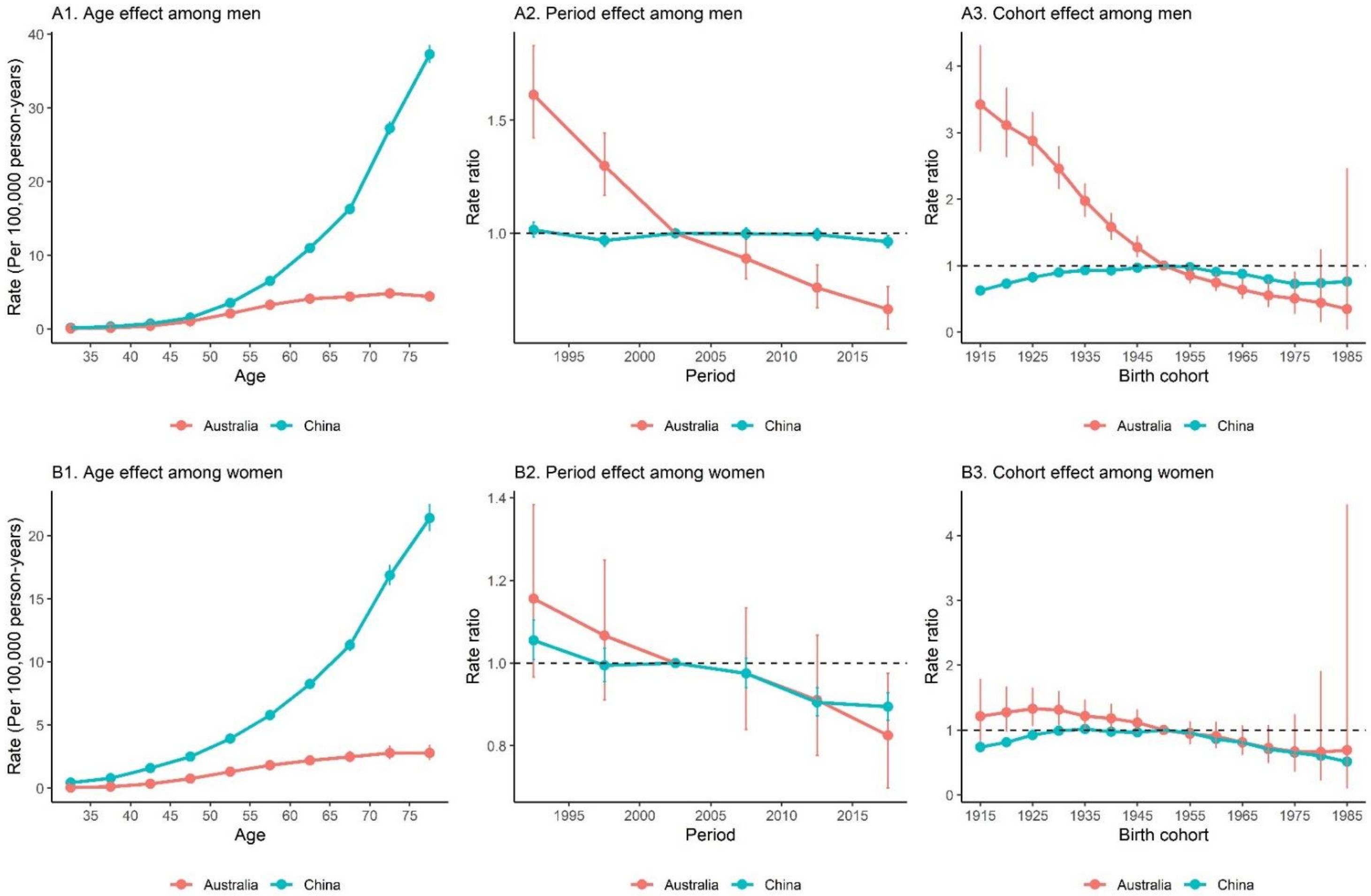

Supplementary Figure S5. Parameter estimates of age, period, and cohort effects on lung cancer mortality attributable to diet low in fruits in China and Australia, 1990 to 2019

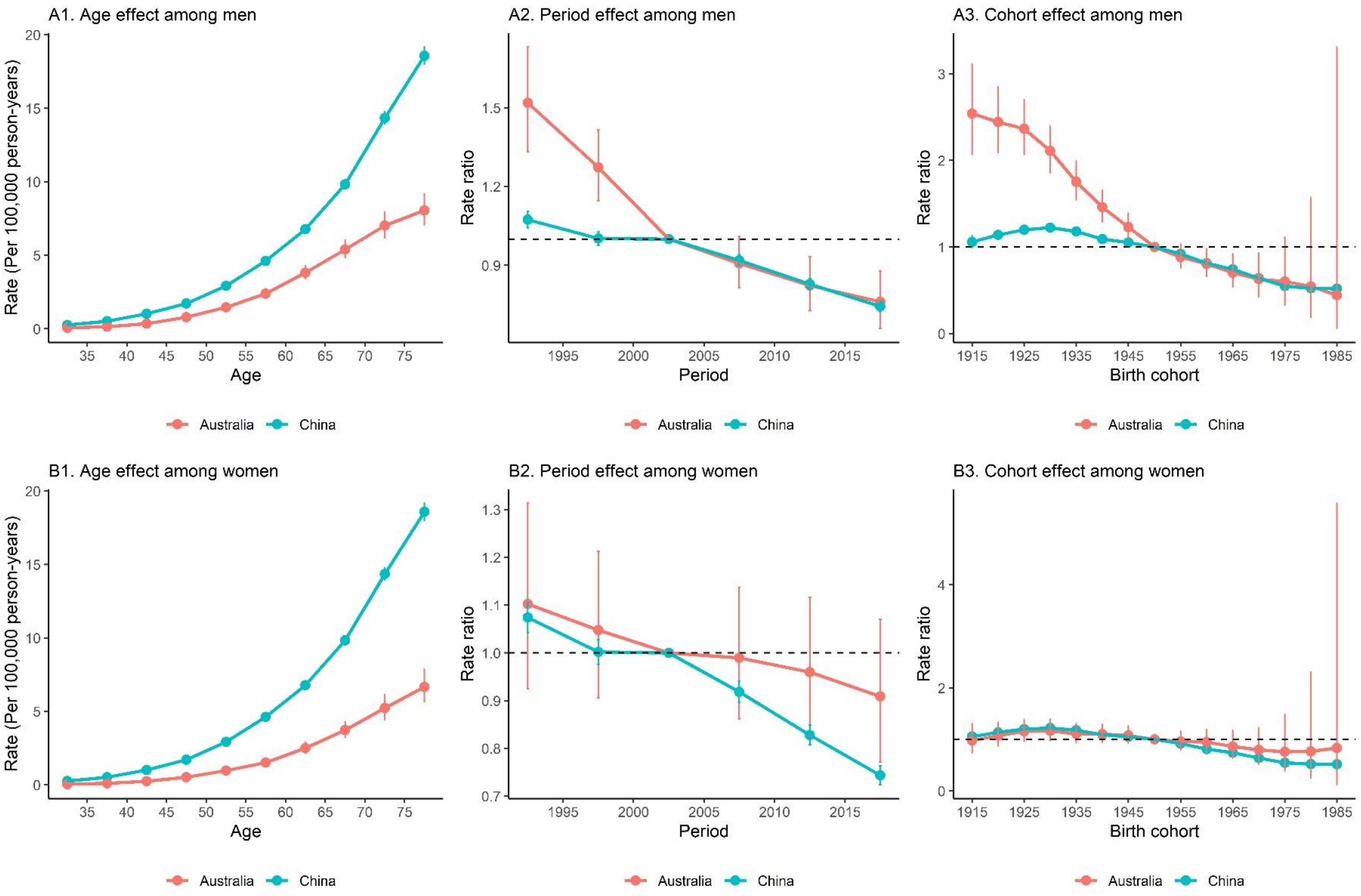

Supplementary Figure S6. Parameter estimates of age, period, and cohort effects on lung cancer mortality attributable to ambient particulate matter in China and Australia, 1990 to 2019

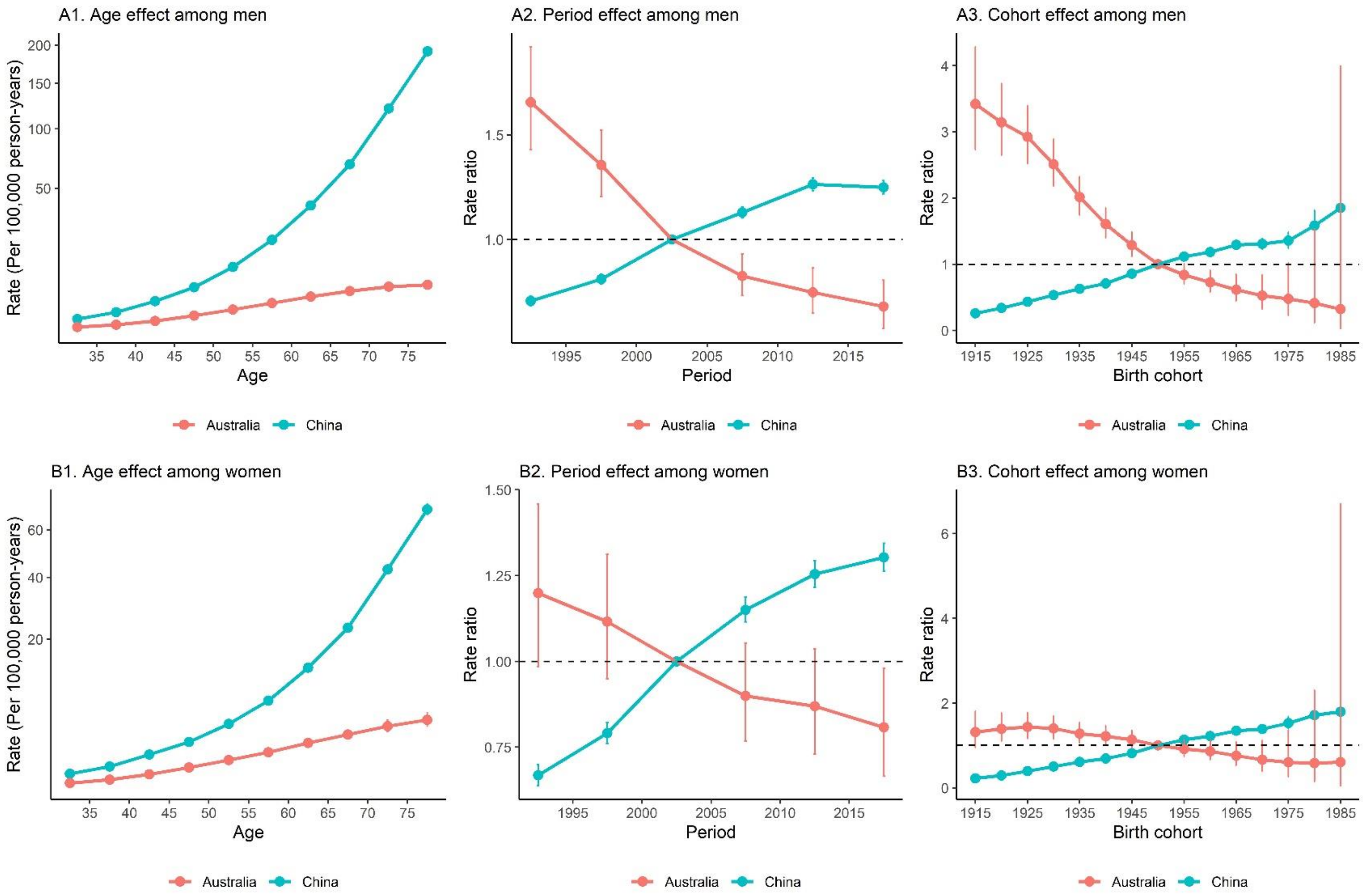

Supplementary Figure S7. Parameter estimates of age, period, and cohort effects on lung cancer mortality attributable to household air pollution from solid fuels in China and Australia, 1990 to 2019

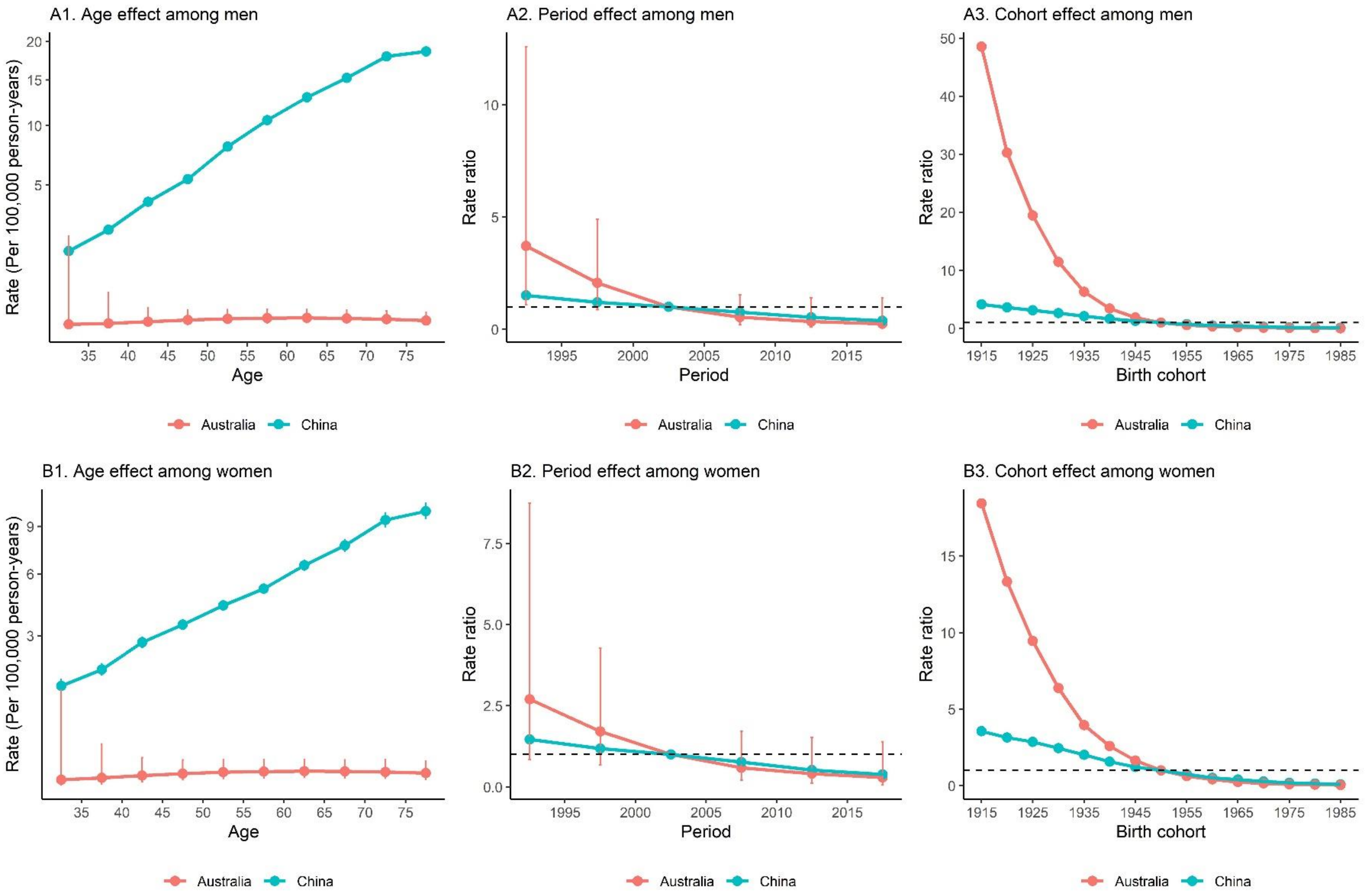

Supplement: Supplementary file 1 — Supplementary Figures. [file 41598_2022_12483_MOESM1_ESM.pdf]
